# Development of a novel imaging system for cell therapy in the brain

**DOI:** 10.1186/s13287-015-0129-7

**Published:** 2015-07-21

**Authors:** Maria-Adelaide Micci, Debbie R. Boone, Margaret A. Parsley, Jingna Wei, Igor Patrikeev, Massoud Motamedi, Helen L. Hellmich

**Affiliations:** Department of Anesthesiology, University of Texas Medical Branch, 301 University Blvd., Galveston, TX 77555 USA; Center for Biomedical Engineering, University of Texas Medical Branch, 301 University Blvd., Galveston, TX 77555 USA

## Abstract

**Introduction:**

Stem cells have been evaluated as a potential therapeutic approach for several neurological disorders of the central and peripheral nervous system as well as for traumatic brain and spinal cord injury. Currently, the lack of a reliable and safe method to accurately and non-invasively locate the site of implantation and track the migration of stem cells *in vivo* hampers the development of stem cell therapy and its clinical application.

In this report, we present data that demonstrate the feasibility of using the human sodium iodide symporter (*hNIS*) as a reporter gene for tracking neural stem cells (NSCs) after transplantation in the brain by using single-photon emission tomography/computed tomography (SPECT/CT) imaging.

**Methods:**

NSCs were isolated from the hippocampus of adult rats (Hipp-NSCs) and transduced with a lentiviral vector containing the hNIS gene. Hipp-NSCs expressing the hNIS (NIS-Hipp-NSCs) were characterized *in vitro* and *in vivo* after transplantation in the rat brain and imaged by using technetium-99m (^99m^Tc) and a small rodent SPECT/CT apparatus. Comparisons were made between Hipp-NSCs and NIS-Hipp-NSCs, and statistical analysis was performed by using two-tailed Student’s *t* test.

**Results:**

Our results show that the expression of the hNIS allows the repeated visualization of NSCs *in vivo* in the brain by using SPECT/CT imaging and does not affect the ability of Hipp-NSCs to generate neuronal and glial cells *in vitro* and* in vivo*.

**Conclusions:**

These data support the use of the hNIS as a reporter gene for non-invasive imaging of NSCs in the brain. The repeated, non-invasive tracking of implanted cells will accelerate the development of effective stem cell therapies for traumatic brain injury and other types of central nervous system injury.

## Introduction

During the past decade, several reports have explored the therapeutic potential of neural stem cells (NSCs) for the treatment of neurological disorders of the central nervous system (CNS) (i.e., macular degeneration, Parkinson’s disease, Alzheimer’s disease, and multiple sclerosis) and for traumatic brain and spinal cord injury [1–8]. However, the successful development of stem cell therapy and its translation to the clinical setting is currently hampered by the lack of a reliable and safe method to accurately monitor the location, migration, and phenotypical differentiation of transplanted cells. So far, the majority of preclinical studies on stem cell fate have relied on *ex vivo* histological analysis of green fluorescent protein (GFP) or β-galactosidase expression in the grafted cells. These analyses require the euthanasia of the animals at each time point studied and therefore are laborious and time-consuming. Recently, the development of *in vivo* non-invasive imaging technologies has provided the means to monitor the delivery, grafting, and survival of stem cells. Current imaging modalities to monitor cells in the brain include magnetic resonance imaging (MRI), single-photon emission tomography (SPECT), positron emission tomography (PET), bioluminescence, and fluorescence imaging [9].

The use of fluorescence and luciferase for bioluminescence imaging is an excellent tool to monitor grafted cells in small animals but is not translatable to the human patient. MRI and SPECT/PET are non-invasive imaging modalities that are suited for human use. Although MRI has a higher spatial resolution than SPECT/PET, the sensitivity of detection is higher for SPECT/PET than MRI (SPECT/PET: 10^−10^–10^−12^ M levels of probe; MRI: 10^−3^–10^−5^ M levels of probe). Additionally, PET has the ability to detect reporter genes [10, 11].

The use of reporter genes to track stem cell fate *in vivo* is particularly appealing, as it is the only method that allows studying stem cell survival (only viable cells will be able to express the reporter protein), proliferation (the reporter gene will be passed on to daughter cells, and the corresponding imaging signals will increase in intensity), and death (cells that are apoptotic or dead will not be able to express the reporter protein). Moreover, a reporter gene can be placed under cell-specific promoters (e.g., neuron-specific or glia-specific), thus allowing monitoring of the fate of the transplanted cells within the host tissue [12, 13].

When clinicians decide on which reporter gene to use for imaging stem cells, important factors to be considered are (1) biological distribution of the gene, (2) availability of the probes, and (3) effect of the expression of the reporter gene on the physiology of the cells.

In this respect, the sodium iodide symporter (NIS) reporter gene represents a good choice for imaging stem cells in the brain because (1) it is not expressed in the brain, (2) the radio probes for NIS are readily available in most nuclear medicine clinics and no radio synthesis is required, (3) the metabolism and clearance in the body of both radiodiodide and technetium-99m (^99m^Tc) are well known, and (4) the imaging potentials of NIS have been shown *in vitro* and *in vivo* [14, 15].

The use of the NIS to monitor *in vivo* the delivery, grafting, and phenotypical differentiation of cells after transplantation has recently been investigated in particular in cardiovascular research [16–18]. The NIS has also been used to monitor trafficking of immune cells *in vivo*. Seo *et al.* reported how transfecting immortalized macrophage cell lines with the hNIS allowed monitoring of their migration toward areas of inflammation *in vivo* in nude mice by using PET imaging [19].

Up until now, however, no studies have been reported on the use of the NIS for imaging NSCs in the brain *in vivo*. Here, we report on the development of the use of the NIS for imaging NSCs in the brain.

## Methods

### Animals

Male Sprague–Dawley rats (200–350 g) were used in all of the experiments described. Experimental protocols were approved by the Institutional Animal Care and Use Committee at the University of Texas Medical Branch, Galveston, in accordance with the guidelines provided by the National Institutes of Health.

### Isolation and *in vitro* expansion of rat hippocampal neural stem cells

Cell culture reagents were obtained from Invitrogen (Carlsbad, CA, USA), except where noted. Adult male Sprague–Dawley rats (200–250 g) were anesthetized with isofluorane (4 % by inhalation) and euthanized by decapitation. The brain was rapidly removed, and the hippocampi were identified and dissected out. The hippocampi from three or four rats were collected into a 50-ml Falcon tube containing sterile Dulbecco’s modified Eagle’s medium/F12 (DMEM/F12) medium with antibiotics (penicillin and streptomycin) and kept on ice. The tissue was minced into small pieces in cold Hanks’ Balanced Salt Solution (HBSS) medium containing 1 mM EGTA in a sterile Petri dish. The tissue pieces were transferred into a sterile 50-ml tube containing HBSS (without Ca^2+^ and Mg^2+)^ with 0.1 % collagenase/dispase, 0.01 % DNase I; 1 ml for 100 mg of tissue) and incubated for 30 min at 37 °C. The tissue was triturated every 10 min by using a sterile disposable 5-ml pipette. At the end of the incubation, the cell suspension was centrifuged at 200×*g* for 5 min at room temperature (RT). The pellet was resuspended in HBSS containing 0.025 % trypsin/ethylenediaminetetraacetic acid and incubated for 10 min at 37 °C. An equal volume of DMEM/F12, 2 mM L-glutamine, 10 % fetal bovine serum (FBS), and antibiotics was added to stop the digestion and the cell suspension was centrifuged at 200×*g* for 5 min. The pellet was resuspended in sterile DMEM/F12 with L-glutamine, 10 % FBS, and antibiotics; triturated by using a 1-ml tip; and centrifuged. This step was repeated two to four times until a single cell suspension was obtained. The cell suspension was filtered through a 70-μm Falcon filter, centrifuged at 200×*g* for 5 min, and resuspended in complete growth medium: neurobasal A medium containing the serum-free supplement B27 (without retinoic acid), 2 mM L-glutamine, 20 ng/ml of epidermal growth factor (EGF), 20 ng/ml of basic fibroblast growth factor-2 (FGF-2), and penicillin/streptomycin. The cells were plated in an uncoated T25 flask at approximately 1×10^6^ cells per 10 ml of complete growth medium. The medium was changed after 24 h and every other day after that.

### Coating of plates with poly-ornithine and laminin

Stock solutions were prepared as follows: poly-L-ornithine (Sigma-Aldrich, St. Louis, MO, USA, catalog number P3655) was reconstituted with sterile distilled water at a concentration of 10 mg/ml, and aliquots were stored at −20 °C; laminin (1 mg/ml; Invitrogen) was stored at −20 °C.

The poly-L-ornithine stock solution was diluted 1:1000 in sterile distilled water to a final working concentration of 10 μg/ml and added to the plates as follow: 1.5 ml/well of six-well plates; 0.2 ml/well of eight-well chambered slides (Lab-Tek II Chambered Slide System; Nalgene Nunc International, Naperville, IL, USA). Plates and slides were incubated overnight at RT and then washed twice with distilled water, incubated with laminin overnight at RT (10 μg/ml in Dulbecco’s phosphate-buffered saline (D-PBS) without Ca^2+^ and Mg^2+^; 1 ml/well of six-well plates and 0.2 ml/well of eight-well chambered slides), and stored at −20 °C until ready to use. The plates were washed with D-PBS once before use.

### Induction of differentiation

Hipp-NSCs were dissociated into single cells by using StemPro Accutase (Invitrogen) in accordance with the instructions of the manufacturer and counted on a hemocytometer. The cells were plated onto poly-ornithine/laminin-coated plates as follows: 125,000 cells per well into a six-well plate and 10,000 cells per well into eight-well chambered slides. To induce differentiation, the cells were cultured for 8–10 days in neurobasal A medium containing B27 with retinoic acid, 0.5 mM L-glutamine, 1 % FBS, and penicillin/streptomycin. The medium was changed every other day.

### Lentivirus packaging

The full-length human sodium iodide symporter cDNA (*hNIS*, a kind gift from Sissy M. Jhiang) was cloned into the pCDH-CMV-MCS-EF1-copGFP cDNA cloning and expression lentivector (System Biosciences, Mountain View, CA, USA). The lentivector product was packaged into VSV-G pseudotyped viral particles by System Biosciences (titer 3.12×10^9^ infectious units/ml).

### Transduction of Hipp-NSCs

Adult rat Hipp-NSCs were dissociated into single cells by using StemPro Accutase (Invitrogen) in accordance with the instructions of the manufacturer and counted on a hemocytometer. The cells were divided into sterile 1.5-ml Eppendorf tubes (100,000 cells per tube in 250 μl of complete growth medium). Pseudovirus particles packaged with the lentivector containing the hNIS were added to each tube at 5, 10, 20, and 40 multiplicity of infection, and the tubes were incubated at 37 °C in a 5 % CO_2_ incubator for 30 min. The cells were then transferred to a 24-well plate (250 μl of cells was added to 750 μl of complete growth medium per well) and placed in a 5 % CO_2_ incubator at 37 °C. After 48 h, the cells were collected into a sterile 15-ml conical tube, centrifuged at 200×*g* for 5 min, resuspended in complete growth medium, and plated out into a sterile T75 flask. The medium was changed every other day. Expression of GFP (indicative of transduction efficiency) was monitored by using an inverted fluorescent microscope equipped with a 488-nm excitation filter (Nikon Eclipse TS100; Nikon, Tokyo, Japan).

### Fluorescence-activated cell sorting

Transduced Hipp-NSCs were dissociated into single cells by using StemPro Accutase, resuspended in sterile D-PBS containing 1 % bovine serum albumin at a concentration of 10–20×10^6^ cells/ml, and passed through a 70-μm Falcon filter. The cells were kept on ice and brought to the University of Texas Medical Branch (UTMB) Flow Cytometry Core Facility, where they were run through a FACSAria cell sorter (BD Biosciences, San Jose, CA, USA). The cells were sorted on the basis of the expression of GFP and collected into a 15-ml tube containing 1 ml of complete growth medium with 10 mM HEPES. The collected cells were centrifuged and replated in fresh complete growth medium.

### *In vitro* uptake of technetium-99m

^99m^Tc was obtained from the Department of Nuclear Medicine at UTMB. Cells (Hipp-NSCs expressing the hNIS and naïve Hipp-NSCs not transduced) were plated out into a six-well plate at a density of 500,000 cells/ml (1.5×10^6^ cells per well) in complete growth medium. The cells were incubated with ^99m^Tc (3–5 μCi/well) for 30 min at 37 °C in a 5 % CO_2_ incubator and then washed with ice-cold PBS two times by collection and centrifugation at 200×*g* for 5 min. At the end of the last centrifugation, the cells were lysed in 500 μl of NaOH (0.33 M) containing 1 % SDS. Counts per minute (CPMs) were read on a gamma counter. Baseline readings were obtained from tubes containing NaOH-1 % SDS and no cells. Data were corrected by subtracting the baseline reading averaged from three separate measurements from the sample CPMs.

### Cell proliferation assay

Cell proliferation was measured by using the CellTiter 96 Aqueous non-radioactive cell proliferation assay (Promega Corporation, Madison, WI, USA). This assay is based on the principle that a tetrazolium compound ([3-(4,5-dimethylthiazol-2-yl)-5-(3-carboxymethoxyphenyl)-2-(4-sulfophenyl)-2H-tetrazolium, inner salt] (MTS)) is converted by viable cells into a soluble formazan product that can be measured by reading the absorbance at 490 nm. Briefly, Hipp-NSCs and Hipp-NSCs transduced with the hNIS were dissociated into single cells by using StemPro Accutase, plated out into a 96-well plate (10,000 and 20,000 cells per well) in complete growth medium (100 μl/well), and cultured for 24–48 h. At the desired time point, 20 μl of MTS/PMS solution (prepared in accordance with the instructions of the manufacturer) was added to each well and the plate was incubated for 3–4 h at 37 °C in a humidified incubator, 5 % CO_2_ atmosphere. The plate was read in an enzyme-linked immunosorbent assay plate reader (Molecular Devices, Sunnyvale, CA, USA) at 490 nm. Background absorbance was measured from wells that were loaded with complete growth medium only and to which MTS/PMS was added.

### Total protein extraction and Western Blot Analysis

Cells growing as neurospheres in complete growth medium were collected in a 15-ml conical tube and centrifuged at 200×*g* for 5 min at RT. The cells were resuspended in D-PBS and centrifuged again. RIPA lysis buffer containing protease inhibitor cocktail, phosphatase inhibitors cocktail, and 100 μM phenylmethylsulfonyl fluoride (PMSF) (all from Sigma-Aldrich) was added to the cell pellet (150–250 μl of lysis buffer for 2–5×10^6^ cells). The lysed cells were transferred to 1.5-ml Eppendorf tubes, vortexed, and incubated on ice for 5 min and then quickly frozen in liquid nitrogen and stored at −80 °C. Cells growing on poly-ornithine/laminin-coated plates were detached by using StemPro Accutase, collected into 15-ml tubes, and centrifuged at 200×*g* for 5 min. The cell pellet was washed in D-PBS and lysed in RIPA lysis buffer as described above. Total protein content was measured by using a Pierce BCA protein assay kit (Thermo Scientific, Rockford, IL, USA).

The protein samples were processed for SDS-polyacrylamide gel electrophoresis (PAGE) with XCell SureLock® Mini-Cell (Invitrogen). After electrophoresis, proteins were transferred to polyvinylidene difluoride (PVDF) membranes (Bio-Rad Laboratories, Hercules, CA, USA) overnight at 4 °C. Blots were incubated in blocking buffer (5 % non-fat dry milk in Tris-buffered saline containing 0.1 % Tween 20; TBS-T) for 1 h at RT and probed with primary antibodies diluted in blocking buffer overnight at 4 °C. After three washes in TBS-T, the blots were incubated with horseradish peroxidase-conjugated secondary antibodies (Cell Signaling Technology, Inc., Danvers, MA, USA; 1:200 dilution in blocking buffer) for 1 h at RT.

Detection was performed using ECL plus kit (Amersham, GE Healthcare, Little Chalfont, UK) followed by exposure of x-ray films (Bioexpress, Kaysville, UT, USA) that were developed with M35A-X-OMAT Processor (Kodak, Rochester, NY, USA). Band intensities were quantified by using ImageJ software, and data were normalized to the expression of the housekeeping protein GAPDH (glyceraldehyde 3-phosphate dehydrogenase).

### Immunofluorescence analysis

Cells growing on poly-ornithine/laminin-coated chambered slides were washed in PBS (pH 7.4) and fixed in 4 % paraformaldehyde (pH 7.4 in PBS) for 15 min at RT. After two washes in PBS, the cells were blocked and permeabilized in PBS containing 5 % normal goat serum and 0.3 % triton X-100 for 30 min at RT. The cells were incubated with primary antibodies (Table 1) diluted in 1.5 % normal goat serum in PBS overnight at 4 °C in a humid chamber. After three washes in PBS, the cells were incubated with secondary antibodies (1:400; Alexa-conjugated anti-mouse and anti-rabbit; Invitrogen) in 1.5 % normal goat serum in PBS for 1 h at RT in a humid chamber. The cells were washed two times in PBS and once in tap water before being coverslipped with mounting medium containing DAPI (4′,6-diamidino-2-phenylindole), a fluorescent stain that binds strongly to A-T-rich regions in DNA and is used to label cell nuclei (Vector laboratories, Burlingame, CA, USA). The slides were viewed with an Olympus BX51 fluorescence microscope equipped with a cooled charge-coupled device camera (Microfire; Optronics, Goleta, CA, USA) and image acquisition software (PictureFrame; Optronics).

**Table 1:** List of antibodies used for the phenotypical characterization of Hipp-NSCs and hNIS-Hipp-NSCs

Primary antibody	Specificity	Dilution	Source
Mouse anti-nestin	Neural progenitor cells	1:5000 WB	Millipore
		1:200 IF	
Rabbit anti-SOX2	Neural progenitor cells	1:2000 WB	Abcam
		1:100 IF	
Mouse anti-βIII-tubulin	Immature and mature neurons	1:5000 WB	Promega
		1:1000 IF	
Mouse anti-NeuN	Mature neurons	1:2000 WB	Millipore
		1:1000 IF	
Rabbit anti-GFAP	Astrocytes	1:5000 WB	Dako
		1:1000 IF	
Mouse anti-oligodendrocyte specific protein	Oligodendrocytes	1:200,000 IF	Millipore
Mouse anti-hNIS	Human NIS	1:100 IF	Abcam

*Hipp-NSC* hippocampus-derived neural stem cell, *hNIS-Hipp-NSC* hippocampus-derived neural stem cell expressing the human sodium iodide symporter, *WB* Western blot, *IF* immunofluorescence, *anti-GFAP* anti-glial fibrillary acidic protein

### Injection of Hipp-NSCs in the hippocampus

Hipp-NSCs were labeled with the cell tracker CMFDA (Invitrogen), suspended at a concentration of 50,000 cells/μl in D-PBS, and kept on ice until ready to inject. Experimental recipient adult male rats were deeply anesthetized with isoflurane (2–4 %) and intubated with an endotracheal tube (6.5-French tubing attached to 16-gauge needle adapter) attached to a ventilator (tidal volume set at 12–15 cc/kg and breath rate set at approximately 28–30 breaths per minute). The anesthetized rats were placed at the flat-skull position on a small animal stereotaxic apparatus, and a craniotomy was performed. The cells were injected by using a 33-gauge cannulae supported by the outside 26-gauge guide cannulae connected with a Hamilton syringe. The cells were injected into the hippocampal CA1—anterior-posterior (AP): −3.6, medial-lateral (ML): +2.0, dorsal-ventral (DV): −2.6—and CA3 (AP: −3.6, ML: +3.6, DV: −3.6) regions (1 μl/site at a speed of 0.2 μl/min). The number of cells injected was chosen on the basis of the work by Gao *et al.* [20].

### Injection of Hipp-NSCs in the lateral ventricles

Hipp-NSCs were suspended in D-PBS at a concentration of 2×10^5^ cells/μl. Experimental recipient adult male rats were deeply anesthetized with isoflurane (2–4 %) and intubated with an endotracheal tube (6.5-French tubing attached to 16-gauge needle adapter) attached to a ventilator (tidal volume set at 12–15 cc/kg and breath rate set at approximately 28–30 breaths per minute). The anesthetized rats were placed at the flat-skull position on a small animal stereotaxic apparatus, and a craniotomy was performed. The injection site was drilled by using a Dremel drill 1.5 mm posterior to bregma and 1.2 mm to lateral right of sagittal suture for ventricular pressure verification, and injection of cells was performed (5 μl; 0.2 μl/min).

### SPECT/CT imaging

Imaging was performed by using an Inveon™ PET-SPECT-CT system (Siemens Medical Solutions, Knoxville, TN, USA) equipped with two gamma cameras (for SPECT modality) and x-ray source and camera for CT imaging mounted on a rotating stage. The PET detector is mounted separately and was not used in this study. This scanner combines PET, SPECT, and CT modalities in one system under the control of a single workstation that allows integrated data acquisition and processing. CT imaging provides mostly anatomical reference information (where the gamma signal comes from), and SPECT provides functional information showing the intensity and the distribution of the isotope in the body. Injected in the animal, a gamma-emitted isotope serves as a source of signal, and the signal is detectable by the SPECT cameras. Since the isotope (in our study, ^99m^Tc) is specifically attaching to the cells with NIS (naturally, to the thyroid cells; in our study, to the modified stem cells) and is not attaching to the other cells, it provides the imaging contrast between the signal (coming from the cells of interest) and background. Cameras are rotated around the animal and the signal collected for 3D reconstruction. In general, we followed the same CT/SPECT imaging as described by Terrovitis *et al.* [16].

### *In vivo* SPECT imaging

The rats were anesthetized with isofluorane and positioned on the bed of the SPECT/CT module with their nose inside a mask connected to the isofluorane dispenser. High-resolution CT scans were performed before each SPECT session. The ^99m^Tc signal was co-registered to the CT scan image by using data processing software Inveon Research Workspace (Siemens Medical Solutions). CT parameters were 2048×3072 pixels, 70 kV, 500 μA, and 520 steps, giving a 3D isotropic resolution of approximately 0.1 mm. SPECT parameters were 40 projections and 9-degree step, for a total of about 16 min for one scan.

### Intracerebral cannulation for delivery of ^99m^Tc in the brain

The rats were anesthetized with isoflurane (2–4 %) and intubated with an endotracheal tube (6.5-French tubing attached to 16-gauge needle adapter) attached to a ventilator (tidal volume set at 12–15 cc/kg and breath rate set at approximately 28–30 breaths per minute). The anesthetized rats were placed at the flat-skull position on a small animal stereotaxic apparatus, and a craniotomy was performed. A microdialysis CMA guide cannula/probe (BASi, West Lafayette, IN, USA) was inserted into the hippocampus (Bregma: −4.3 mm; ML: −3.5 mm; Dura: −3.2 mm).

### Tissue processing and immunofluorescence analysis

At the desired time point after cell transplantation, the rats were anesthetized with pentobarbital and cardially perfused with heparinized saline followed by ice-cold 4 % paraformaldehyde (pH 7.4). The brains were removed and embedded in 20 % sucrose in PBS (pH 7.4). Sections (20 μm) were cut on a cryostat, collected on glass microscope slides (Superfrost Plus; Thermo Fisher Scientific Inc., Marietta, OH, USA), and stored at −20 °C. For immunofluorescence analysis of grafted cells, the sections were hydrated in PBS and incubated in PBS containing 10 % normal goat serum and 0.3 % Triton X-100 for 30 min at RT. The sections were incubated with primary antibodies diluted in PBS containing 1.5 % normal goat serum, overnight at 4 °C and with secondary antibodies (594 Alexa-conjugated, Invitrogen; 1:400 dilution in PBS with 1.5 % normal goat serum) for 1 h at RT. After washing in PBS, the sections were rinsed in tap water and coverslipped with mounting medium with DAPI (Vector Laboratories).

### Statistical analysis

Data were expressed as the mean ± standard error. Statistical analysis between two independent experimental groups was performed by using two-tailed Student’s *t* test. Results were considered significant for *P* values of less than 0.05.

## Results

### Expansion and characterization of Hipp-NSCs

**Fig. 1:**
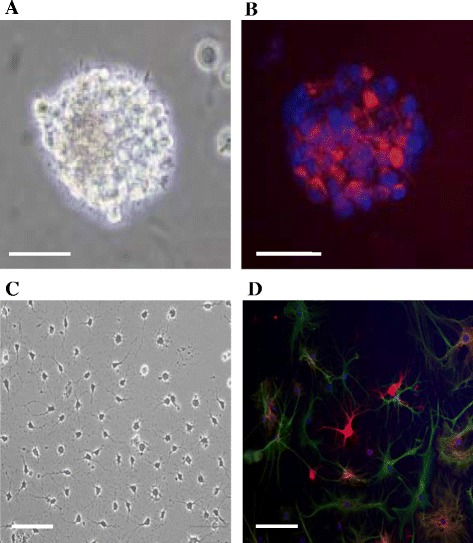
Hipp-NSCs form neurospheres and generate neurons and glia *in vitro*. **a** A typical neurosphere formed by Hipp-NSCs growing in serum-free medium with EGF and FGF-2. **b** Immunofluorescence analysis of nestin expression (red) in Hipp-NSC neurospheres. Nuclei are stained blue with DAPI. **c** Phase-contrast photograph of Hipp-NSCs growing on poly-ornithine-coated plates in differentiation medium for 8 days. **d** Immunofluorescence analysis of Hipp-NSCs after 8 days of differentiation showing the expression of the neuronal marker βIII-tubulin (red) and of the glial marker GFAP (green). Nuclei are stained blue with DAPI. Scale bars = 50 μm. *DAPI* 4′,6-diamidino-2-phenylindole, *EGF* epidermal growth factor, *FGF-2* fibroblast growth factor-2, *GFAP* glial fibrillary acidic protein, *Hipp-NSC* hippocampus-derived neural stem cell

NSCs were isolated from the hippocampus of adult rats and maintained in culture in serum-free neurobasal A medium supplemented with B27 and the growth factors EGF and FGF-2 (complete growth medium). Under these conditions, the cells actively proliferate and form a characteristic spheroid group of cells that express the NSC marker nestin (Fig. 1a, b). NSCs are defined not only by their ability to proliferate and generate daughter cells that are unspecialized and express specific stem cell markers but also by their ability to generate neurons and glia. When Hipp-NSCs were plated onto poly-ornithine/laminin-coated plates in serum-free medium containing B27, retinoic acid, and no growth factors (EGF and FGF-2), they attached to the substrate and underwent rapid morphological changes (Fig. 1c). Immuncytochemical analysis of cells grown for 8 days under differentiating conditions confirmed that both neurons and glia were generated from Hipp-NSCs (Fig. 1d).

### Generation of Hipp-NSCs expressing the hNIS

**Fig. 2:**
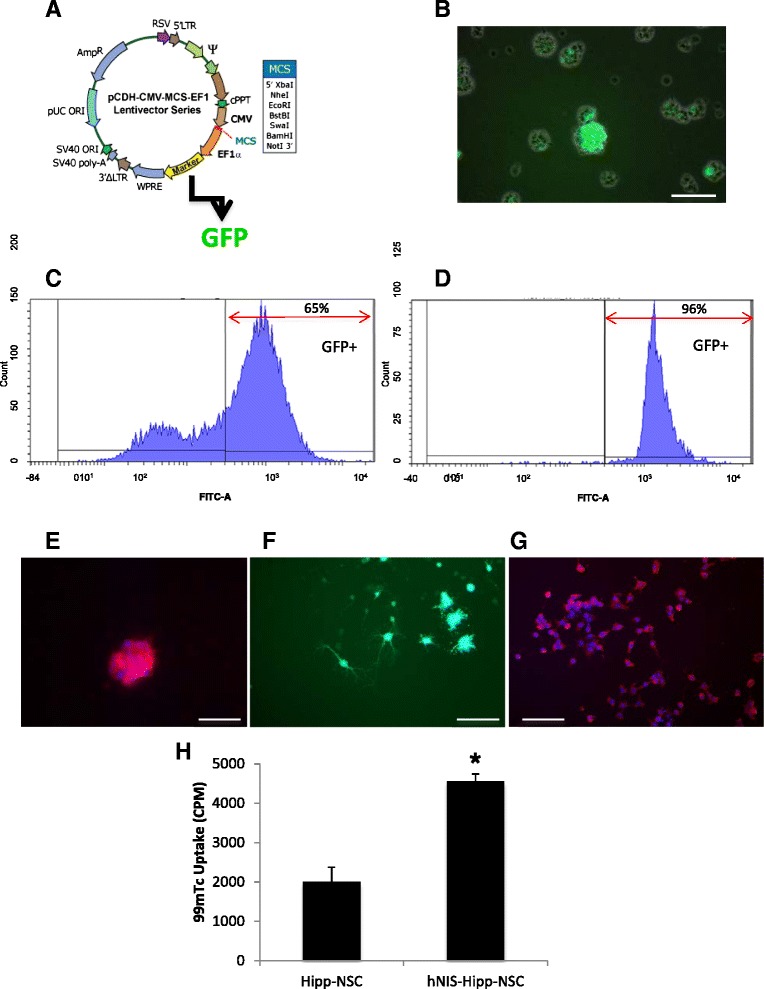
Generation of Hipp-NSCs expressing the hNIS. **a** Map of the HIV-based lentivector CD11B-1 (System Biosciences). The *hNIS* cDNA was cloned in the multiple cloning site (MSC) located downstream of the CMV promoter. Downstream of the expression cassette for the hNIS, an EF1 promoter drives the expression of GFP. **b** Expression of GFP in Hipp-NSCs transduced with pseudoviral particles for 48 h. The fluorescent image is superimposed on the phase-contrast image. **c** Flow cytometric analysis of Hipp-NSCs transduced with the hNIS showing the expression of GFP (indicative of the expression of hNIS) in 62 % of the cells before sorting and in 96 % of the cells after sorting (**d**). **e** Immunofluorescence analysis of the expression of the hNIS (in red) in Hipp-NSCs transduced with the lentivector and selected by sorting for GFP. Nuclei are stained blue with DAPI. **f** Expression of GFP in differentiated Hipp-NSCs transduced with the hNIS. **g** Immunoreactivity for the hNIS in differentiated NIS-Hipp-NSCs. Nuclei are counterstained blue with DAPI. **h** Uptake of ^99m^Tc in NIS-Hipp-NSCs was measured as counts per minute (CPM) in a gamma counter and corrected by subtracting background CPM. *N* = 3; *P < 0.01 by Student’s *t* test. Scale bars = 100 μm. *CMV* cytomegalovirus, *DAPI* 4′,6-diamidino-2-phenylindole, *GFP* green fluorescent protein, *Hipp-NSC* hippocampus-derived neural stem cell, *hNIS* human sodium iodide symporter, *NIS-Hipp-NSC* hippocampus-derived neural stem cell expressing the human sodium iodide symporter, ^*99m*^ *Tc* technetium-99m

Hipp-NSCs were transduced by using pseudoviral particles packaged with a lentivector containing the hNIS and the reporter gene GFP (Fig. 2a, b). The expression of GFP and fluorescence-activated cell sorting was used to select out Hipp-NSCs expressing the hNIS (Fig. 2c, d). The expression of the hNIS in the sorted cells was confirmed by immunocytochemical analysis (Fig. 2e). We also determined whether the expression of the hNIS would be retained after differentiation. Hipp-NSCs transduced with the hNIS were cultured onto poly-ornithine/laminin-coated plates in differentiation medium for 7 days. The hNIS was maintained in the differentiated cells as shown by the expression of GFP and by immunofluorescence analysis by using a specific antibody against hNIS (Fig. 2f, g).

To determine whether the hNIS expressed by Hipp-NSCs is functional, we tested the ability of the transduced cells to transport ^99m^Tc, a gamma-emitting radioisotope that is used by the NIS instead of endogenous iodide. A significant increase in CPM was measured in Hipp-NSCs expressing the hNIS as compared with control Hipp-NSCs not expressing the hNIS and exposed to ^99m^Tc (Fig. 2h).

### Characterization of Hipp-NSCs expressing the hNIS *in vitro*

**Fig. 3:**
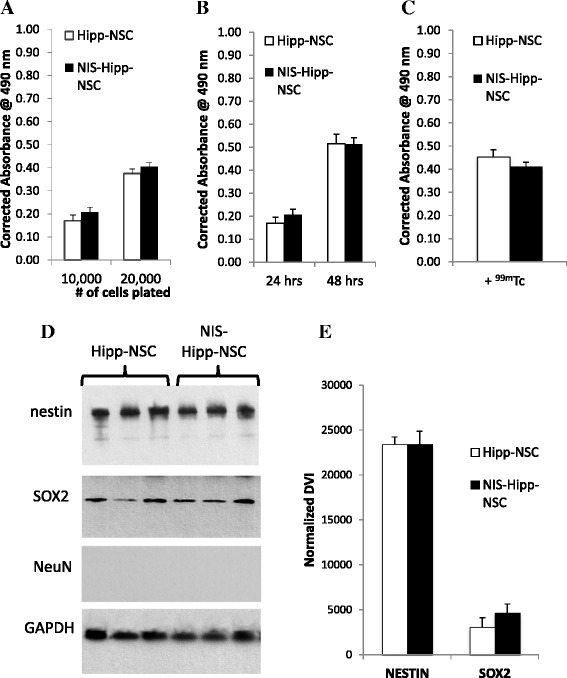
NIS-Hipp-NSCs are viable, proliferate, and express NSC markers. MTS-based analysis of cell viability (**a**) and proliferation (**b**) of Hipp-NSCs transduced with the *hNIS* (NIS-Hipp-NSCs) compared with naïve Hipp-NSCs. *N* = 3; *P* > 0.05 by Student’s *t* test. **c** Hipp-NSCs and NIS-Hipp-NSCs were exposed to ^99m^Tc for 30 min. MTS-based analysis of cell viability was performed 48 h later. *N* = 3; *P* > 0.05 by Student’s *t* test. **d** Western blot analysis of total protein extracts from NIS-Hipp-NSCs and naïve Hipp-NSCs grown under proliferating conditions. **e** Densitometric analysis. The expression of GAPDH was used to normalize the densitometry value index (DVI). *N* = 3; *P* > 0.05 by Student’s *t* test. *GAPDH* glyceraldehyde 3-phosphate dehydrogenase, *Hipp-NSC* hippocampus-derived neural stem cell, *hNIS* human sodium iodide symporter, *MTS* 3-(4,5-dimethylthiazol-2-yl)-5-(3-carboxymethoxyphenyl)-2-(4-sulfophenyl)-2H-tetrazolium, inner salt, *NIS-Hipp-NSC* hippocampus-derived neural stem cell expressing the human sodium iodide symporter, *NSC* neural stem cell, ^*99m*^ *Tc* technetium-99m

To assess the effect of the hNIS on cell viability, Hipp-NSCs were plated in a 96-well plate in neurobasal A medium containing EGF and FGF (complete growth medium) at two different densities (10,000 and 20,000 cells per well) and cultured for 24 h. To measure proliferation, the cells were plated out at a density of 10,000 cells per well and cultured for 24 and 48 h. Cell viability was assessed by using the MTS assay (CellTiter 96 Aqueous non-radioactive cell proliferation assay; Promega Corporation). No significant differences were detected between Hipp-NSCs expressing the hNIS (NIS-Hipp-NSCs) and Hipp-NSCs in their naïve form (not expressing the hNIS) (Fig. 3a, b). To test the effect of ^99m^Tc incorporation on cell viability, Hipp-NSCs and NIS-Hipp-NSCs were exposed to ^99m^Tc (100 μCi added to the cell culture medium) for 30 min. The cells were washed, resuspended in proliferation medium, plated out in a 96-well plate (10,000 cells per well), and cultured for 48 h. Cell viability was assessed 48 h later by using the MTS assay (CellTiter 96 Aqueous non-radioactive cell proliferation assay). No significant differences were detected between Hipp-NSCs and NIS-Hipp-NSCs, indicating that the incorporation of ^99m^Tc in NIS-Hipp-NSCs does not reduce cell viability. In addition to studying the effect of the hNIS on Hipp-NSC viability and proliferation, we studied whether NIS-Hipp-NSCs retain their characteristic stem cell signature. This was done by analyzing the expression of NSC markers by Western blot analysis. There was no difference in the expression of nestin and SOX2 between Hipp-NSCs transduced with the hNIS (NIS-Hipp-NSCs) and naïve Hipp-NSCs (Fig. 3c, d).

### Differentiation potential of Hipp-NSCs expressing the hNIS

**Fig. 4:**
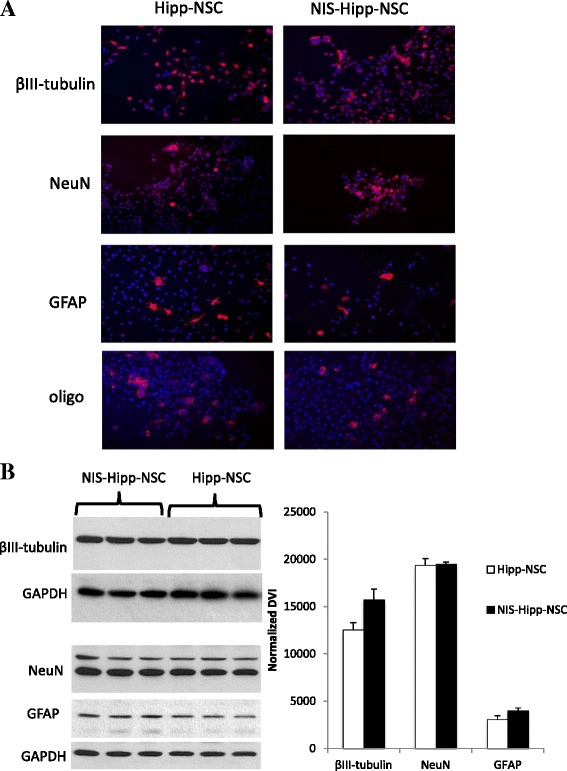
Differentiation potential of NIS-Hipp-NSCs. **a** Immunofluorescence analysis of Hipp-NSCs transduced with the *hNIS* (NIS-Hipp-NSCs) and of naïve Hipp-NSCs cultured in differentiation medium for 7 days. Nuclei are counterstained blue with DAPI. **b** Western blot analysis of total protein extracts from differentiated NIS-Hipp-NSCs and naïve Hipp-NSCs. The expression of GAPDH was used to normalize the densitometry value index (DVI). *N* = 3; *P < 0.05 by Student’s *t* test. *DAPI* 4′,6-diamidino-2-phenylindole, *GAPDH* glyceraldehyde 3-phosphate dehydrogenase, *Hipp-NSC* hippocampus-derived neural stem cell, *hNIS* human sodium iodide symporter, *NIS-Hipp-NSC* hippocampus-derived neural stem cell expressing the human sodium iodide symporter

To test the effect of hNIS on the ability of Hipp-NSCs to generate neurons and glia, the cells were plated out onto poly-ornithine/laminin-coated plates and cultured for 8 days in neurobasal medium containing B27 with retinoic acid and 1 % FBS (differentiation medium). Neuronal and glial differentiation was assessed by immunofluorescence and Western blot analysis. The results show that the expression of hNIS does not affect the ability of Hipp-NSCs to generate neurons and glia *in vitro* (Fig. 4).

### SPECT/CT imaging of Hipp-NSCs expressing the hNIS

#### In vitro imaging

**Fig. 5:**
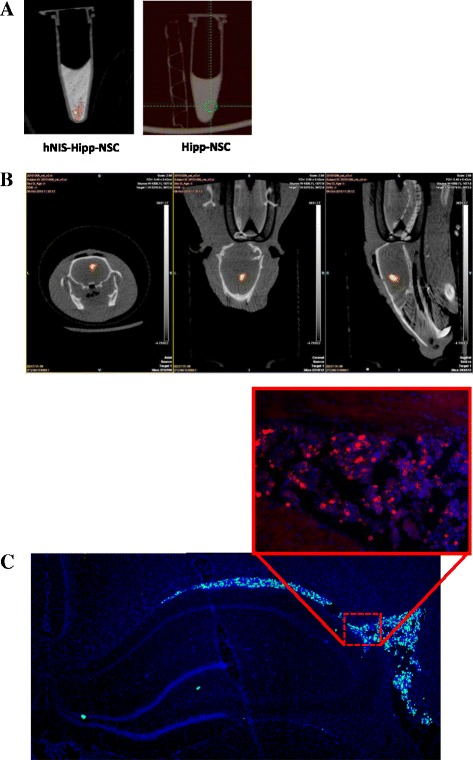
SPECT imaging of NIS-Hipp-NSCs. **a** SPECT signal from hNIS-Hipp-NSCs and Hipp-NSCs that were incubated with ^99m^Tc for 30 min and washed in phosphate-buffered saline before imaging. The SPECT signal is shown superimposed on the CT scan image. **b** SPECT signal recorded from NIS-Hipp-NSCs that were incubated with ^99m^Tc *in vitro* before transplantation in the right lateral ventricle. Transverse, coronal, and sagittal orientations of the brain are shown. **c** Analysis of the brain shows grafted cells (identified by the expression of GFP) in the ventricle. The expression of the NIS in the grafted cells was confirmed by immunofluorescence by using an antibody against hNIS (*inset*). *CT* computed tomography, *GFP* green fluorescent protein, *Hipp-NSC* hippocampus-derived neural stem cell, *hNIS* human sodium iodide symporter, *NIS* sodium iodide symporter, *NIS-Hipp-NSC* hippocampus-derived neural stem cell expressing the human sodium iodide symporter, *SPECT* single-photon emission tomography, ^*99m*^ *Tc* technetium-99m

Hippocampus-derived NSCs can be visualized by using SPECT imaging by providing ^99m^Tc to the cells and measuring the gamma-ray emission from the cells that have taken up the ^99m^Tc via the NIS. Hipp-NSCs stably expressing the NIS were incubated *in vitro* with ^99m^Tc and imaged by placing the cells in an Eppendorf tube inside the SPECT/CT imaging module. Control cells (Hipp-NSCs not expressing the NIS) were also incubated with ^99m^Tc and imaged (Fig. 5a).

#### In vivo imaging

**Fig. 6:**
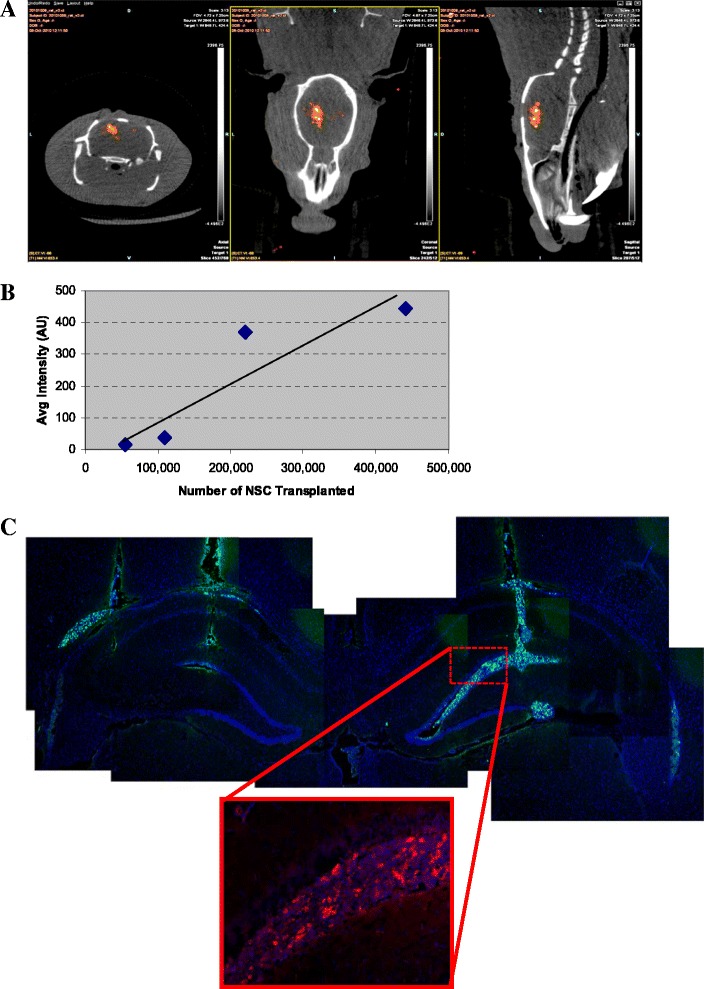
*In vivo* SPECT imaging of NIS-Hipp-NSCs injected in the brain hippocampus. **a** SPECT signal recorded from NIS-Hipp-NSCs that were incubated with ^99m^Tc *in vitro* before transplantation in the hippocampus (two sites/hippocampus). Transverse, coronal, and sagittal orientations of the brain are shown. **b** Correlation between the number of cells injected in the hippocampus and the signal detected by SPECT imaging. Correlation coefficient r = 0.9566; two-tailed *P* value = 0.0108. **c** Analysis of the brain shows grafted cells (identified by the expression of GFP) in the hippocampus. The expression of the NIS in the grafted cells was confirmed by immunofluorescence by using an antibody against hNIS (*inset*). *GFP* green fluorescent protein, *Hipp-NSC* hippocampus-derived neural stem cell, *hNIS* human sodium iodide symporter, *NIS* sodium iodide symporter, *NIS-Hipp-NSC* hippocampus-derived neural stem cell expressing the human sodium iodide symporter, *SPECT* single-photon emission tomography, ^*99m*^ *Tc* technetium-99m

To assess whether grafted cells can be visualized by using SPECT/CT imaging in the live animal, in a first set of experiments we incubated NIS-Hipp-NSCs *in vitro* with ^99m^Tc before transplantation in the rat brain. The cells were injected in the left lateral ventricle (1×10^6^ cells) (Fig. 5b) or in the hippocampus (two injection sites per hippocampus; amount of cells per site ranging from 440,000 to 55,000 cells) (Fig. 6a), and the rats were subjected to SPECT imaging. The SPECT signal was superimposed to the CT scan to localize the cells within the brain. Quantification of the signal in the hippocampus was performed by measuring the average number of pixels in the region of interest by using image analysis software (Image J) and correlated to the number of cells injected (Fig. 6b). Post-imaging analysis of the rat brains confirmed the presence of the cells by immunofluorescence analysis (Fig. 6c).

**Fig. 7:**
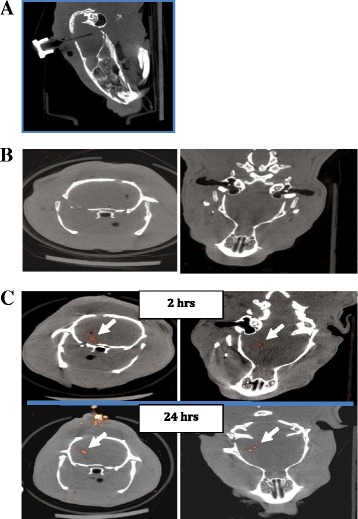
Repetitive *in vivo* SPECT imaging of NIS-Hipp-NSCs. **a** CT scan of the rat head showing the placement of the cannula used to deliver ^99m^Tc into the brain. **b** SPECT imaging of the brain of a rat that had received ^99m^Tc and no cells. **c** SPECT signal recorded from the rat brain 2 and 24 h after NIS-Hipp-NSC intracranial injection following delivering of ^99m^Tc. Transverse and coronal slice orientations are shown. *CT* computed tomography, *NIS-Hipp-NSC* hippocampus-derived neural stem cell expressing the human sodium iodide symporter, *SPECT* single-photon emission tomography, ^*99m*^ *Tc* technetium-99m

In a second set of experiments, NIS-Hipp-NSCs were injected through an intracranial cannula implanted in the rat brain. For these experiments, NIS-Hipp-NSCs were resuspended in PBS and injected in the brain (1–2×10^5^ cells in 5 μl at 0.2 μl/min). At various time points after cell injection (2 and 24 h), ^99m^Tc was delivered to the brain via the cannula (0.5 mCi in 5 μl at a speed of 0.5 μl/min), and the rats were subjected to SPECT/CT imaging 4 h later (Fig. 7).

These data demonstrate that Hipp-NSCs expressing the hNIS can be visualized *in vivo* in the rat brain. Specifically, these data show adequate spatial resolution and signal detection sensitivity of the SPECT imaging system and the feasibility to repetitively image grafted NSCs in the rat brain.

### Characterization of Hipp-NSCs expressing the hNIS after transplantation in the rat brain

**Fig. 8:**
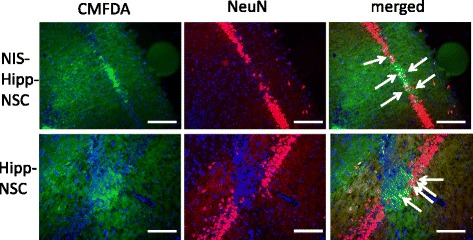
*In vivo* SPECT imaging of NIS-Hipp-NSCs injected in the brain hippocampus. Representative cross-sections of rat brain 2 weeks after transplantation of NIS-Hipp-NSCs or Hipp-NSCs in the hippocampus (CA1 region). Grafted cells are located on the basis of the expression of the cell tracker dye CMFDA. Mature neurons are identified on the basis of the expression of NeuN. Merged images show that some of the grafted cells express the neuronal marker NeuN (*arrows*). Cell nuclei are stained blue with DAPI. Scale bar = 50 μm. *DAPI* 4′,6-diamidino-2-phenylindole, *Hipp-NSC* hippocampus-derived neural stem cell, *NIS-Hipp-NSC* hippocampus-derived neural stem cell expressing the human sodium iodide symporter, *SPECT* single-photon emission tomography

To determine whether the expression of the hNIS would alter the ability of Hipp-NSCs to graft and survive after transplantation in the intact rat brain, Hipp-NSCs expressing the hNIS were injected in the hippocampus of adult rats and the animals were euthanized 2 weeks later. Control groups consisted of rats that were injected with Hipp-NSCs without the hNIS. Grafted cells in the brain were localized on the basis of the presence of CMFDA (a cell tracker dye that was loaded in the cells before transplantation). Both Hipp-NSCs and Hipp-NSCs expressing the hNIS were found grafted in the hippocampus 2 weeks after transplantation. Grafted cells were localized in the pyramidal layer (CA1) and some cells were also visible between the CA1 and granular layer. Phenotypical characterization of the grafted cells performed by immunofluorescence analysis revealed that some of the grafted cells had differentiated into mature neuronal cells expressing NeuN (Fig. 8).

## Discussion

### NIS expression in NSCs

The first objective of this work was to determine the effect of NIS expression on the biology of NSCs. The analysis of the effect of the expression of NIS on Hipp-NSCs was directed to both the basic biological properties as well as to their ability to respond to the host brain tissue *in vivo*. Our data show that the expression of NIS in Hipp-NSCs does not change their proliferation rate *in vitro*. The ability to proliferate is a key functional signature of NSCs and is of particular importance in clinical applications because *in vitro* NSC expansion is necessary to generate a clinically useful number of cells for transplantation. Biochemical expression of proteins that are characteristically found in NSCs (SOX2 and nestin) was also not affected by the NIS. Although the expression of SOX2 and nestin is commonly used to identify NSCs, a more complete analysis of their biochemical profile and of their karyotype should be performed in future studies. Nonetheless, the results shown in this work demonstrate that the exogenous expression of NIS in Hipp-NSCs does not alter the basic biological properties of the cells. This is in agreement with other studies that have characterized the use of the NIS in other types of stem cells.

Possibly the most important feature of NSCs, and one that makes these cells so appealing for use in replacement therapy of the nervous system, is their ability to generate neurons and glia. This important feature is maintained in Hipp-NSCs that express the NIS, as demonstrated by the expression of proteins that are known markers of differentiated phenotypes—βIII-tubulin and NeuN for neurons and glial fibrillary acidic protein (GFAP) and oligodendrocyte-specific protein for glia—analyzed by using both immunofluorescence and Western blot techniques. Additionally, no differences were found between Hipp-NSCs expressing the NIS and naïve Hipp-NSCs when comparing the expression of neuronal and glial markers by Western blot analysis. This is true both *in vitro* as well as *in vivo* in the rat brain, where Hipp-NSCs expressing the NIS where found grafted in the hippocampus 2 weeks after transplantation with at least some cells showing expression of neuronal markers. Although the studies presented in this report are limited to a 2-week follow-up, a large body of literature testifies to the safety of ^99m^Tc accumulation in cells *in vivo* [16, 21–23]. Specifically, NIS-based imaging has been validated and used in humans [24, 25], thus supporting the translational potential of this reporter system.

### Molecular imaging of NSCs using the NIS

The main goal of this work was to develop an imaging system that will allow tracking NSCs after transplantation in the brain. We have provided several pieces of evidence, *in vitro* and *in vivo*, that the expression of the reporter gene hNIS allows the visualization of live and viable Hipp-NSCs by using SPECT imaging. We have first demonstrated that the expression of the hNIS in Hipp-NSCs is functional in that it allows for the selective uptake of ^99m^Tc inside the cells. ^99m^Tc is a gamma-emitting radioisotope that is taken up by the NIS in place of iodide (the endogenous, biological ligand of the NIS). Moreover, ^99m^Tc is widely available and currently approved by the US Food and Drug Administration in combination with SPECT imaging for diagnostic purposes.

In the *in vivo* experiments, ^99m^Tc was delivered directly in the brain parenchyma where the cells were grafted. The results demonstrate that the Hipp-NSCs grafted in the brain are viable, can take up ^99m^Tc via the NIS, and can be visualized by SPECT imaging.

A successful imaging system needs to have sufficient signal sensitivity to detect grafted cells and sufficient spatial resolution to determine their location within the tissue/organ. The data presented in this article demonstrate that NIS combined with SPECT/CT imaging possesses enough spatial resolution and sensitivity to detect viable grafted cells in the brain. Specifically, when Hipp-NSCs were injected at different sites within the hippocampus and at different densities, discrete regions of positive signals were detected and a correlation between the signal intensity and the number of cells injected could be derived. Another important result of this work is the demonstration that Hipp-NSCs can be visualized repetitively over time on the same rat. This is a critical aspect of a reporter gene imaging system because, by allowing the visualization of grafted cells over time and for as long as they are viable, it sets this system apart from other imaging methodologies.

### Limitations

One limitation of using the hNIS reporter system for imaging cells in the brain parenchyma is the relatively low permeability of ^99m^Tc through the blood–brain barrier (BBB). In this report, therefore, we opted to use an intracranial cannula to deliver ^99m^Tc directly to the brain in order to bypass any potential issue with ^99m^Tc penetration through the BBB. This allowed us to provide proof-of-principle data demonstrating the feasibility of tracking grafted NSCs in the brain by using the NIS reporter system and SPECT detection. More clinically relevant and non-invasive delivery routes for ^99m^Tc have been reported, including intravenous injections to image intracranial gliomas in experimental animals [26, 27], injection into the olfactory route [28, 29], encapsulation of ^99m^Tc into liposomes, or nanoparticles targeted to the CNS via specific BBB transporters or receptor systems [30–32]. In future studies, we will test these routes of delivery; however, even as it stands, our data show that delivery of ^99m^Tc via implanted intracranial cannulas is a valuable method for tracking NSCs in the brain in preclinical animal models of disease that will allow researchers to follow cell grafts over time in the same animals. This not only will provide valuable information on the location and viability of the cells and their biological activity but also will significantly shorten the time and reduce the number of experimental animals needed to perform the studies, thus expediting the translation of stem cell therapy to the clinical setting.

Although here we used NSCs isolated from the hippocampus of adult rats (Hipp-NSCs), it has to be noted that more easily accessible sources of NSCs (i.e., induced pluripotent stem cells, embryonic stem cell-derived NSCs, and NSCs derived from mesenchymal stem cells isolated from bone marrow, fat, or umbilical cord) have been described and are clinically attractive sources of cells as they allow autologous transplantation, an appealing approach that eliminates the need for immunosuppressive therapies. Because NSCs share common basic biological properties, the results of this work are significant, as they lay the groundwork for the use of the NIS to image NSCs and can very likely be extrapolated to other sources of NSCs.

Additionally, it is important to notice that in this study the cells were transplanted in naïve/uninjured rats. The response of Hipp-NSCs to an injured (i.e., after traumatic brain injury) or diseased brain will likely differ. However, a detailed analysis of grafting and differentiation potentials of Hipp-NSCs in the injured and uninjured brain goes beyond the scope of this work and will be the focus of future studies.

## Conclusions

In this report, we have characterized the NIS as a reporter gene for imaging NSCs in the brain. Developing non-invasive imaging of grafted cells has emerged as a fundamental tool to advance the field of stem cell therapy. The ability to track cells *in vivo* after transplantation on the same animal over time will allow a reduction in the number of experimental animals needed to determine the optimal source of cells as well as the best route and site of delivery that will produce a functional recovery in animal models of disease. This will significantly shorten the time between experimental animal work and translation to the clinical setting.

## Abbreviations

AP: Anterior-posterior
BBB: Blood–brain barrier
CNS: Central nervous system
CPM: Counts per minute
CT: Computed tomography
DAPI: 4′,6-diamidino-2-phenylindole
DMEM: Dulbecco’s modified Eagle’s medium
D-PBS: Dulbecco’s phosphate-buffered saline
DV: Dorsal-ventral
EGF: Epidermal growth factor
FBS: Fetal bovine serum
FGF-2: Fibroblast growth factor-2
GFP: Green fluorescent protein
HBSS: Hanks’ Balanced Salt Solution
Hipp-NSC: Hippocampus-derived neural stem cell
hNIS: Human sodium iodide symporter
ML: Medial-lateral
MRI: Magnetic resonance imaging
MTS: 3-(4,5-dimethylthiazol-2-yl)-5-(3-carboxymethoxyphenyl)-2-(4-sulfophenyl)-2H-tetrazolium, inner salt
NIS: Sodium iodide symporter
NIS-Hipp-NSC: Hippocampus-derived neural stem cell expressing the human sodium iodide symporter
NSC: Neural stem cell
PBS: Phosphate-buffered saline
PET: Positron emission tomography
PMS: Phenazine methosulfate
SPECT: Single-photon emission tomography
TBS-T: Tris-buffered saline containing 0.1 % Tween 20
Tc: Technetium-99m
UTMB: University of Texas Medical Branch
